# Outcome Measures of Open versus Minimally Invasive Surgery for Thoracolumbar Spinal Traumatic Fractures: A Systematic Review and Meta-Analysis

**DOI:** 10.3390/jcm13185558

**Published:** 2024-09-19

**Authors:** Felice Esposito, Ilaria Bove, Francesca Vitulli, Andrea Bocchino, Andrea Barbanera, Stefania Nape, Sara Lombardi, Giovanni Raffa, Luigi Pintore, Carmela Palmiero, Fabrizio Fellico, Domenico Solari, Luigi Maria Cavallo, Teresa Somma

**Affiliations:** 1 Department of Neurosciences and Reproductive and Dental Sciences, Division of Neurosurgery, Federico II University of Naples, 80131 Naples, Italy; ilariabove90@gmail.com (I.B.); vitullifrancesca@gmail.com (F.V.); andreabocchino1994@gmail.com (A.B.); stefania.nape@live.it (S.N.); lombasara3@gmail.com (S.L.); luigipintore8@gmail.com (L.P.); carme.palmiero@gmail.com (C.P.); fabriziofellico@gmail.com (F.F.); domenico.solari@unina.it (D.S.); lcavallo@unina.it (L.M.C.); teresa.somma85@gmail.com (T.S.); 2 Division of Neurosurgery, Academic Hospital of Alessandria, 15121 Alessandria, Italy; abarbanera@ospedale.al.it; 3 Department of Biomedical, Dental and Imaging Sciences, Division of Neurosurgery, University of Messina, 98100 Messina, Italy; giovanni.raffa@unime.it

**Keywords:** pedicle screw fixation, percutaneous stabilization, thoracolumbar fracture, pain, outcome, meta-analysis

## Abstract

**Objective:** To evaluate the efficacy of open and percutaneous pedicle screw fixation in the treatment of thoracolumbar fractures. **Methods:** Online databases MEDLINE (PubMed), SCOPUS, and Cochrane were searched for English language articles published between January 2001 and December 2023, limited to articles that included the clinical and radiological outcomes of adult patients. The main outcome measures of the study were the Oswestry Disability Index (ODI), the Numeric Rating Scale (NRS) score, and the Cobb angle. **Results:** A total of 7 studies involving 909 patients were included; 374 (41.1%) procedures were performed with open surgery (OS), while 535 (58.9%) procedures were conducted with minimally invasive surgery (MIS). The mean value of ODI in the MIS group was 8.29% [CI 95% 4.82–11.76], compared to the other group, which was 14.22% (*p*-value 0.87). Patients receiving a MIS had an average NRS of 1.54 [CI95% 0.98–2.10] whilst OS had 2.31 [CI95% 1.50–3.12] (*p*-value 0.12). **Conclusions:** The percutaneous technique is equally safe and effective in resolving the deformity, but the clear advantages are represented by the reduction in blood loss, shorter operative times, a lower incidence of infection, shorter hospitalization, shorter postoperative rehabilitation, and therefore good results in terms of quality of life.

## 1. Introduction

Traumatic fractures (TFs) are the most common spinal injuries, with a prevalence of 50% to 70%. The thoracolumbar junction $\left(T_{12}-L_{2}\right)$ (TL) represents the most affected segment, with an annual incidence of 13 to 30 cases/100,000 [1]. Several studies confirmed a slight prevalence in men (62%). The most frequent etiologies are represented by traffic accidents (38.7%) and falls (23.8%) [1]. Symptoms and signs can vary: they can be a consequence of alteration of the vertebral balance in the coronal and sagittal planes (that is, post-traumatic deformity) or spinal cord compression [2].

The treatment of TL fractures can be conservative or surgical. The choice of treatment depends on the site, the type of fracture, and the clinical status. Surgical treatment mainly involves posterior fixation to restore spinal stability and eventually decompress neural elements. The use of open pedicle screw fixation is a well-established technique; on the other hand, a wide range of minimally invasive surgery (MIS) (i.e., vertebroplasty, kyphoplasty, or percutaneous pedicle screw placement) has been widely used during the last decade [3]. The percutaneous pedicle screw placement avoids soft tissue and muscle stripping, reduces intraoperative blood loss, and allows a shorter postoperative hospitalization [4,5]. The efficacy of postoperative pain, quality of life, and spinal deformity has been widely discussed in the literature and, to date, there is no common consensus on the best strategy to adopt [6]. The study aims to report a meta-analysis of the literature analyzing the clinical and radiological outcomes in patients with traumatic fractures of the thoracolumbar junction (thoracolumbar fractures—TLFs) treated with the two different approaches, that is, posterior open versus percutaneous stabilization.

## 2. Materials and Methods

Online databases MEDLINE (PubMed), EMBASE, and Cochrane were searched for English language articles published between 2001 and 2022 containing the following keywords alone or in combination: “Outcome(s) after open spinal stabilization”, OR “Outcome(s) after spinal percutaneous stabilization”, OR “Outcome(s) after spinal percutaneous vs. open stabilization”, OR “Spinal open stabilization”, OR “Spinal percutaneous stabilization”, OR “Spinal open stabilization complication(s)”, OR “Spinal percutaneous stabilization complication(s)”, OR “Open stabilization complication(s)”, OR “Percutaneous stabilization complication(s)”, OR “Open stabilization”, OR “Percutaneous stabilization”.

Comparison studies eligible for this systematic review and meta-analysis included those in which the authors compared percutaneous instrumentation approaches with open pedicle screw techniques for TFLs. When institutions published duplicate studies with the accumulation of patient numbers, they were excluded for quantitative evaluation at each time interval. All publications were limited to those involving human subjects and in English. Abstract, case reports, lecture presentations, editorials, reviews, and expert opinions were excluded.

The abstracts were reviewed, and each article of interest was marked for further review. The full texts of the marked studies were retrieved, and the studies that met the inclusion criteria were included in this analysis. The references listed in each article were reviewed to identify pertinent articles. Case reports were excluded from the study. For centers that published multiple studies on patients described in previous reports, the most recent and/or comprehensive study was used.

With such a methodology, of the initial 13,714 articles retrieved, 8885 were duplicate records or records marked as ineligible by automation tools or removed for other reasons; 4421 articles were excluded after the title/abstract screen, and 404 + 197 were excluded after the full text screen and data extraction. Finally, 7 articles were analyzed, having satisfied the inclusion criteria.

The comparative studies eligible for the systematic review and meta-analysis included those in which patient cohorts compared percutaneous instrumentation approaches with open pedicle screw approaches for TFLs. When the same institution published duplicate studies with the accumulation of patient numbers, they were excluded for quantitative evaluation at each time interval. All publications were limited to those involving human subjects and in the English language. Abstract, case reports, lecture presentations, editorials, reviews, and expert opinions were excluded.

### 2.1. Data Extraction and Analysis

The following variables were extracted: number of patients treated, type of surgery, ODI score, NRS score, Cobb angle, and complications. The main outcome measures of the study were the Oswestry Disability Index (ODI) score, the Numeric Rating Scale (NRS) score, and the Cobb angle.

The modified German version of the Oswestry Disability Index (ODI) was used to evaluate spinal function and pain as spine-specific outcome criteria [7]. The Numeric Rating Scale (NRS) is a pain screening tool commonly used to assess the severity of pain using a scale of ‘zero to ten’ (0–10), where ‘zero’ means ‘no pain’ and ‘ten’ means ‘the worst pain imaginable’ [8]. The Cobb angle is the most widely used measurement to quantify the magnitude of spinal deformities on plain radiographs. The Cobb angle technique can assess the degree of kyphosis or lordosis in the sagittal plane.

Data from these articles were pooled to calculate the baseline characteristics. Two reviewers (A.B. and I.B.) independently performed the literature search and data extraction. Discrepancies in data extraction were resolved by the consensus of both reviewers.

### 2.2. Statistical Analysis

Raw data were entered into Microsoft Excel (Version 10.14, Mac). Statistical analyses were performed with R (version 4.0.2; The R Foundation for Statistical Computing) and RStudio (version 1.2.1335), with *p* < 0.05 considered statistically significant. Demographic characteristics and outcomes between MIS and open surgery (OS) were compared using the ‘meta mean’ function in R. Whether random effects or fixed effects should be used was determined by the I^2^ tests. The “Forest” function in R was used for the forest plot with subgroup analysis. Heterogeneity was formally assessed using Q, I^2^, and τ^2^ statistics. As recommended by the Cochrane Statistics Methods Group, I^2^ was interpreted as follows: 0–40% low heterogeneity; 30–60% moderate heterogeneity; 50–90% substantial heterogeneity; and 75–100% considerable heterogeneity.

To examine publication bias, funnel plot analysis was performed using the “funnel” function in R.

### 2.3. Compliance to PRISMA Guidelines

The present meta-analysis is compliant with PRISMA guidelines. The full PRISMA checklist can be found in the Supplementary Materials of the manuscript.

### 2.4. Systematic Review Registration

The present study was not registered in any systematic review registry.

## 3. Results

**Figure 1:**
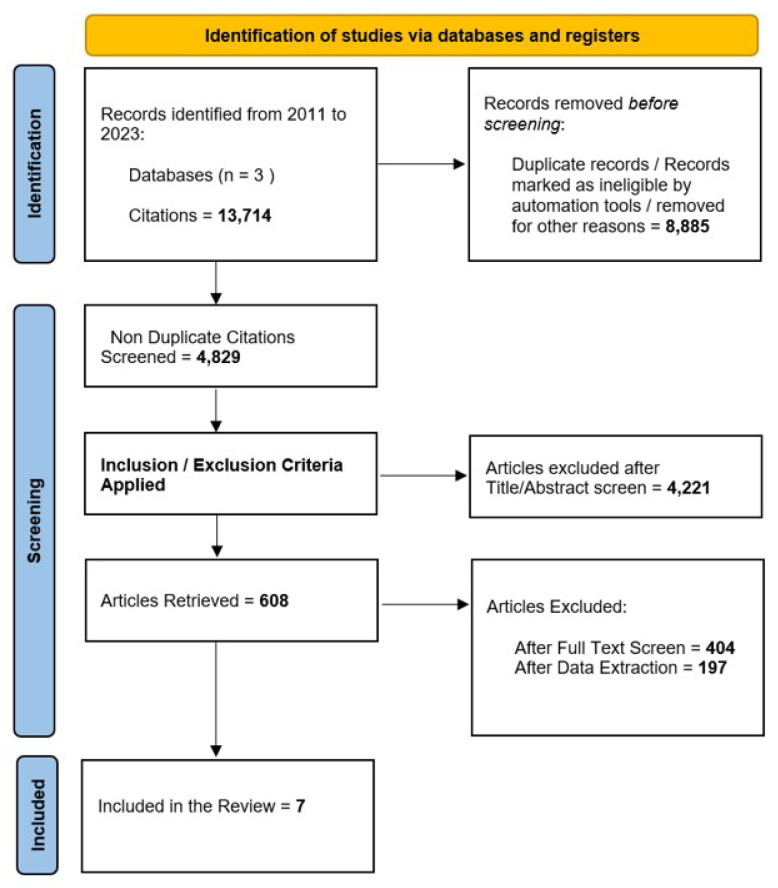
PRISMA flowchart showing the process involved in identifying relevant literature.

After screening 13,714 articles, 7 were analyzed, having satisfied our inclusion criteria (Figure 1).

Six were retrospective cohort studies [3,9,10,11,12,13], while only one was a prospective study [14].

The baseline characteristics for 909 patients are summarized in Table 1. The data analysis of all features are summarized in Table 2.

**Figure 2:**
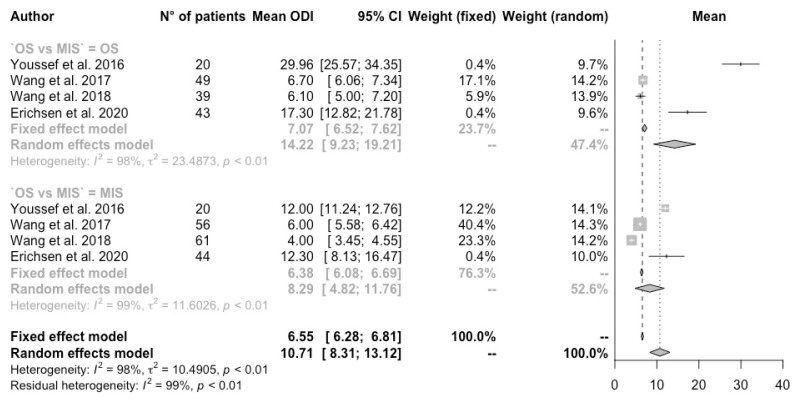
Forest plot of the ODI score [3,11,13,14]. Subgroup meta-analysis.

A total of 374 (41.1%) procedures were performed with OS, while 535 (58.9%) procedures were performed with MIS (*p*-value 0.99). All features were analyzed using a random-effects model. The ODI, NRS, and Cobb angle were not significantly different between the two groups. In particular, the mean value of ODI in the MIS group was 8.29% [CI 95% 4.82–11.76], compared to the other group, which was 14.22% [CI 95% 9.23–19.21] (*p*-value 0.87) (Figure 2).

**Figure 3:**
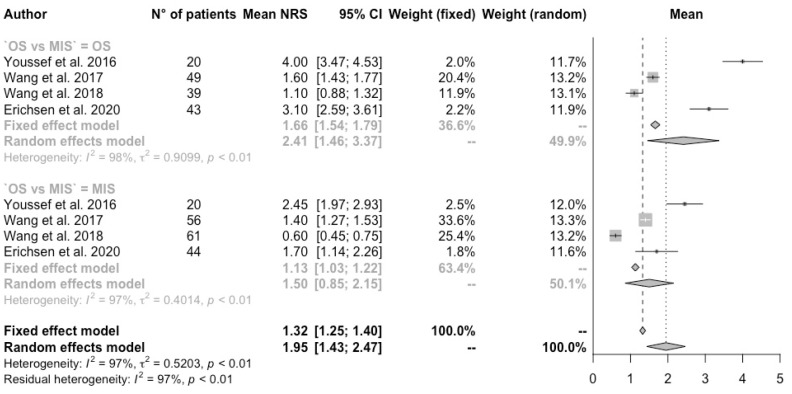
Forest plot of NRS values [3,11,13,14]. Subgroup meta-analysis.

Patients receiving a MIS had an average NRS of 1.54 [CI 95% 0.98–2.10], whilst OS had 2.31 [CI 95% 1.50–3.12] (*p*-value 0.12) (Figure 3).

**Figure 4:**
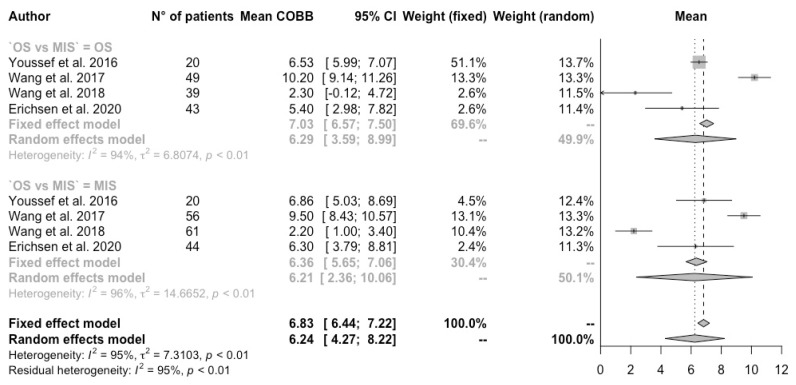
Forest plot of Cobb angle [3,11,13,14]. Subgroup meta-analysis.

The Cobb angle was 6.82 [CI 95% 3.56–10.08] in MIS patients and 7.00 in OS [CI 95% 4.64–9.36] (*p*-value 0.92) (Figure 4).

The test for heterogeneity revealed considerable heterogeneity between studies, with an I^2^ statistics of 99%, τ^2^ of 10.49 and a *p*-value < 0.01 for ODI; I^2^ statistics of 97%, τ^2^ of 0.48 and a *p*-value < 0.01 for NRS; I^2^ of 94%, τ^2^ of 7.01, and a *p*-value < 0.01 for the Cobb angle.

**Figure 5:**
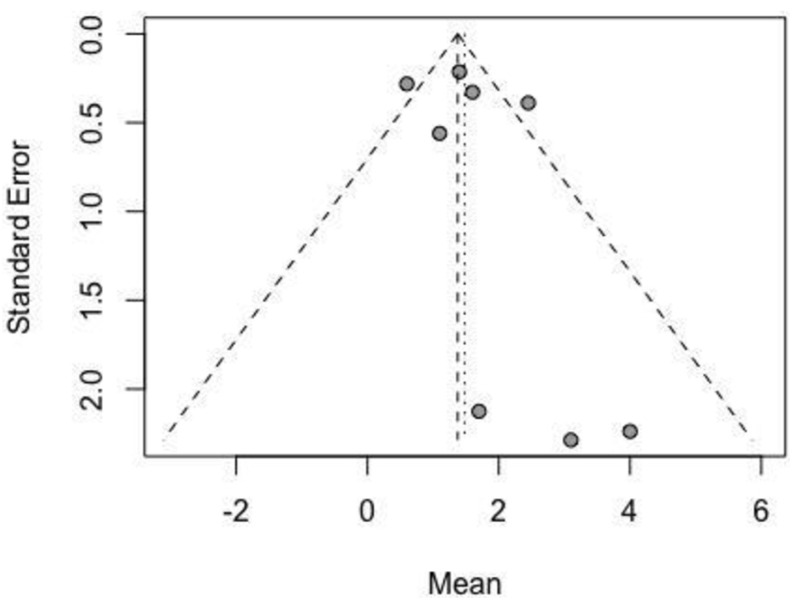
Funnel analysis of publication bias.

The funnel plot analysis showed the absence of publication bias in the selected literature due to a symmetrical funnel and the presence of all articles inside the triangle area (Figure 5).

## 4. Discussion

Thoracolumbar vertebral fractures are a common neurosurgical pathology in clinical practice, with an annual incidence of approximately 30 per 100,000 [1]. Falls and road accidents are the most common causes of such events. Incomplete burst fractures are the most common. Patients may present with a wide range of symptoms and signs, including intense pain with or without neurological signs, and radiological deformity with or without sensory/motor deficits.

Over time, several classifications have attempted to describe the severity of the damage, the degree of instability, and consequently the risk of neurological injury [15]. Magerl classification [16] was adopted as the original AO classification (Arbeitsgemeinschaft für Osteosynthesefragen [Association of Osteosynthesis], shortened to AO) in 1994 and was based on the concept of three columns by Denis [17] and McAfee [18] classification, but was considered too complex and not indicated to guide clinical decisions [19]. Afterwards, in 2013, Divi et al. [20] published the AO-Spine thoracolumbar spine injury classification system to provide treatment recommendations for a wide variety of thoracolumbar injuries. The classification system separates fractures into three major types: Type A (compression injuries), Type B (tension band injuries), and Type C (translational injuries). Type A and B injuries are further subdivided into five and three subtypes, respectively. A surgical algorithm was developed by Rajasekaran et al. [21] in 2017 to complete the AO-Spine thoracolumbar spinal injury classification system. This system proposes conservative treatment for A1–A2 fractures and surgical options for unstable fractures (A3, B, C). For type A2–A3 fractures, the choice between conservative and operative treatment strategy is based on the primary evaluation of the fracture stability, degree of deformity, and clinical state of the patient. Fracture types B2–B3 or C are characterized by the failure of the posterior column to resist distraction, the disruption of the posterior tension banding system, and dislocation. Generally, they require an open approach, while percutaneous fixation is primarily recommended for fracture types A and B1, which are more stable. Sebaaly et al. [22] suggest that the use of the percutaneous technique is limited in cases where there are highly unstable fractures with translational mechanisms (fracture type C and B2 with ligament injuries) and in cases of neurological deficits where open posterior decompression is required. However, several authors indicate that percutaneous fixation is generally sufficient for treating type A and B1 thoracolumbar fractures (AO Spine classification) with good results of kyphosis reduction compared to open instrumentation and fusion and open fixation [11,23,24].

Our comparative study focused on type A fractures, which are more stable and can be treated using open and percutaneous techniques.

Our meta-analysis included data from 909 patients. Of these, 535 (58.85%) were treated with a minimally invasive percutaneous technique and 374 (41.15%) with an open technique. There was no uniformity regarding the type of fractures considered and the classification used (two type A fracture studies [AO-spine classification], two Denis studies, and one Magerl classification).

The open transpedicular approach to thoracolumbar fractures was introduced by Roy-Camille in the 1960s, and since then, an increasingly widespread approach has been seen as an effective strategy to improve symptoms and quality of patient life [25]. The open transpedicular approach allows a wide exposure of the vertebral body, spinal decompression when required, lower radiation exposure, and a short learning curve for spinal surgeons. However, several potential limitations have emerged: the open technique can lead to extensive intraoperative blood loss, a longer operating time, and an increased risk of infection. As suggested by Mobbs et al. [26], dissection of the paraspinal muscle during open spine surgery could cause muscle denervation and increased intramuscular pressure with a high risk of ischemia, resulting in muscle atrophy and scarring. These aspects may be associated with prolonged postoperative pain, increasing the risk of failed back surgery syndrome.

To overcome such limitations, minimally invasive surgery (MIS) techniques have been utilized. Magerl et al. [16,27] introduced the pedicle screw procedure using the percutaneous method. Since then, this technique has been used increasingly. Compared to the open technique, the percutaneous technique requires a steep learning curve and does not allow wide exposure to the operating field or tactile perception of the surgical act. Furthermore, Phan et al. [27] showed how increased exposure to fluoroscopy or radiological guidance is an important limitation of this technique. Despite the initial skepticism, the percutaneous technique has become increasingly popular, and it has been observed that shorter operating times, reduced intraoperative and postoperative blood loss, shorter hospitalization, improved functional results in terms of NRS and reduction in infection rates are the main advantages of the technique [28].

In our study, no significant differences were found in terms of the functional and radiological outcomes between the two branches of treatment; patients reported the same postoperative ODI score, NRS, and Cobb angle (primary functional parameter). The same result was obtained for the radiological results: the postoperative reduction in Cobb angle (primary radiological parameter) was statistically similar between the two groups. Erichsen et al. [3] and Kreinest et al. [12] reported similar results. No significant differences were found in the radiological and clinical outcomes (NRS, ODI, and SF-36) [29]. In contrast, the authors found a shorter operative time in the percutaneous group [3] and a shorter hospitalization with a reduction in intraoperative blood loss, generally below 300 mL [12].

The percutaneous technique shows results comparable to those of the classic open technique in resolving the deformity, but the clear advantages are represented by the reduction in blood loss, shorter operative times, lower incidence of infection, shorter hospitalization, shorter postoperative rehabilitation, and, therefore, good results in terms of quality of life [10].

In addition, the placement of the percutaneous pedicle screw allows less traction of the paravertebral musculature with a lower probability of pain during the postoperative course. These aspects could represent key elements in the treatment of complicated patients, such as the elderly and polytraumatized patients, especially in the case of anticoagulant therapy.

## 5. Study Limitations

Our study has some limitations: (i) this meta-analysis is based on reviewing the clinical information collected from the patients instead of prospectively designing and conducting the study. Six of the seven studies were retrospective. This may have caused selection bias; (ii) we focused our analysis on clinical and radiological outcomes, not considering the influence of other concomitant pathologies (osteoporosis, diabetes, etc.) and a prognostic evaluation. This could have made the data heterogeneous; (iii) we know that single arm studies may lead to several biases but, at the same time, there has been an increasing number of meta-analyses using single-arm studies over the past few years. As a matter of fact, the use of real-world data (RWD) from nonrandomized studies (e.g., single-arm studies) is increasingly being explored to overcome issues associated with data from randomized controlled trials (RCTs). We thought that this might have been our case, as there is a lack of RCTs directly comparing open and percutaneous fusion techniques for the management of vertebral fractures. Either under a frequentist framework, which assumes exchangeability for baseline treatment effects and a common relative treatment effect, and under a Bayesian framework, which assumes exchangeability for treatment effects on each arm, it is possible to combine data from RCTs and single-arm studies at the aggregate level and provide a consistent reduction in uncertainty when including single-arm data whilst remaining robust to data [30,31]. The studies included in our meta-analysis, as the result of a progressive selection of different studies available in the pertinent literature, focus on the open or percutaneous fusion technique. These two treatments have the same objectives: restoration of the vertebral stability, correction of the imbalance, and decompression of the nervous structures. According to the PRISMA method, only seven studies met our inclusion criteria.

## 6. Conclusions

The results of our meta-analysis demonstrated that the use of open pedicle screw fixation and percutaneous pedicle screw placement in the treatment of thoracolumbar fractures is equally safe and effective in terms of clinical, functional, and radiological outcomes. The clear advantages of the percutaneous technique are the reduction in blood loss, shorter operative times, lower incidence of infection, shorter hospitalization, shorter postoperative rehabilitation, and, therefore, good results in terms of quality of life. From this point of view, this technique should be considered the treatment of choice in the treatment of elderly and/or polytraumatized patients, especially in the case of anticoagulant therapy. Future prospective studies are required with more patients and longer follow-up periods.

## Figures and Tables

**Table 1:** Literature summary—study characteristics.

N°	Author	Year	Type of Stydy	Classification	N° of Cases	Pathology	OS	MIS	Technique
1	Fisher et al. [9]	2009	Retrospective	Denis	27	TLFs (T1-T5), unstable	27	-	Posterior OS fixation
2	Lee et al. [10]	2013	Retrospective	Denis	59	TLFs, burst	27	32	short OS—short PPSF without fusion
3	Youssef et al. [14]	2016	Prospective	AO spine	40	TLFs (only type A)	20	20	OS-MIS
4	Kreinest et al. [12]	2016	Retrospective	Magerl’s	491	TLFs	169	322	OS-MIS
5	Wang et al. [11]	2017	Retrospective	N/A	105	TLFs	49	56	OS-MIS
6	Wang et al. [13]	2018	Retrospective	N/A	100	TLFs	39	61	OS-MIS
7	Erichsen et al. [3]	2020	Retrospective	AO spine	87	Traumatic (T11-L2)	27	44	OS (USS *)-MIS (monoaxial [=40] or polyaxial [=4]

* USS: Universal Spine System.

**Table 2:** Literature summary—study results.

N°	Author	ODI-MIS	ODI-OS	NRS-MIS	NRS-OS	Cobb Angle-MIS	Cobb Angle-OS	Complications-MIS	Complications-OS
1	Fisher et al. [9]	-	-	-	-	-	8.7	15	15
2	Lee et al. [10]	-	-	1.70	1.90	9.3	9.9	1	3
3	Youssef et al. [14]	12.00	29.96	2.45	4.00	6.86	6.53	-	-
4	Kreinest et al. [12]	-	-	-	-	-	-	-	-
5	Wang et al. [11]	6.00	6.70	1.40	1.60	9.5	10.2	1	2
6	Wang et al. [13]	4.00	6.10	0.60	1.10	2.2	2.3	2	2
7	Erichsen et al. [3]	12.30	17.30	1.70	3.10	6.3	5.4	-	-

## Data Availability

Not applicable since no new data were created in conjunction with this study.

## Supplementary Materials

The following supporting information can be downloaded at: https://www.mdpi.com/article/10.3390/jcm13185558/s1. Supplementary Materials file: PRISMA 2020 Main Checklist.

## Abbreviations

The following abbreviations are used in this manuscript:

TL	ThoracoLumbar
TLFs	ThoracoLumbar Fractures
TFs	Traumatic Fractures
ODI	Oswestry Disability Index
NRS	Numeric Rating Scale
OS	Open Surgery
MIS	Minimally invasive Surgery
N/A	Not Applicable
AO	Arbeitsgemeinschaft für Osteosynthesefragen (Association of Osteosynthesis)

## References

[1] Zileli M., Sharif S., Fornari M. (2021). Incidence and Epidemiology of Thoracolumbar Spine FracturesWFNS Spine Committee Recommendations. Neurospine.

[2] Roer N.V.D., Lange E.S.M.D., Bakker F.C., Vet H.C.W.D., Tulder M.W.V. (2005). Management of traumatic thoracolumbar fractures: A systematic review of the literature. Eur. Spine J..

[3] Erichsen C.J., Heyde C.E., Josten C., Gonschorek O., Panzer S., Rüden C.V., Spiegl U.J. (2020). Percutaneous versus open posterior stabilization in AOSpine type A3 thoracolumbar fractures. BMC Musculoskelet. Disord..

[4] Hayoun T., Siboni R., Ohl X., Bredin S. (2023). Treatment of thoracolumbar fractures: Comparison of the clinical and radiological outcomes of percutaneous versus open surgery. Eur. J. Orthop. Surg. Traumatol..

[5] Ricciardi L., Stifano V., Proietti L., Perna A., Pepa G.M.D., Rocca G.L., Olivi A., Polli F.M. (2018). Intraoperative and Postoperative Segmental Lordosis Mismatch: Analysis of 3 Fusion Techniques. World Neurosurg..

[6] Huang Q.S., Chi Y.L., Wang X.Y., Mao F.M., Lin Y., Ni W.F., Xu H.Z. (2008). Comparative percutaneous with open pedicle screw fixation in the treatment of thoracolumbar burst fractures without neurological deficit. Zhonghua Wai Ke Za Zhi/Chin. J. Surg..

[7] Osthus H., Cziske R., Jacobi E. (2006). Cross-cultural adaptation of a German version of the Oswestry Disability Index and evaluation of its measurement properties. Spine.

[8] Nugent S.M., Lovejoy T.I., Shull S., Dobscha S.K., Morasco B.J. (2021). Associations of Pain Numeric Rating Scale Scores Collected during Usual Care with Research Administered Patient Reported Pain Outcomes. Pain Med..

[9] Fisher C., Singh S., Boyd M., Kingwell S., Kwon B., Meng J.L., Dvorak M. (2009). Clinical and radiographic outcomes of pedicle screw fixation for upper thoracic spine (T1-5) fractures: A retrospective cohort study of 27 cases. Clinical article. J. Neurosurg. Spine.

[10] Lee J.K., Jang J.W., Kim T.W., Kim T.S., Kim S.H., Moon S.J. (2013). Percutaneous short-segment pedicle screw placement without fusion in the treatment of thoracolumbar burst fractures: Is it effective?: Comparative study with open short-segment pedicle screw fixation with posterolateral fusion. Acta Neurochir..

[11] Wang H., Zhou Y., Li C., Liu J., Xiang L. (2017). Comparison of Open Versus Percutaneous Pedicle Screw Fixation Using the Sextant System in the Treatment of Traumatic Thoracolumbar Fractures. Clin. Spine Surg..

[12] Kreinest M., Rillig J., Grützner P.A., Küffer M., Tinelli M., Matschke S. (2017). Analysis of complications and perioperative data after open or percutaneous dorsal instrumentation following traumatic spinal fracture of the thoracic and lumbar spine: A retrospective cohort study including 491 patients. Eur. Spine J..

[13] Wang X.I., Liu Y., Wang X., Chen H., Cao P., Tian Y.E., Wu X., Chen Y.U., Yuan W.E. (2018). Beneficial effects of percutaneous minimally invasive surgery for patients with fractures in the thoracic spine. Exp. Ther. Med..

[14] Mohammad ElSayed Youssef E., Abd-AlSalam AbdAlFattah H., Hassanin Mohammad S., Abd-AlHamid Daoud E. (2016). Percutaneous Versus Open Pedicle Screw Fixation of Thoracic and Lumbar Fractures; Comparative Study at Zagazig University Hospitals. Br. J. Sci..

[15] Domenicucci M., Preite R., Ramieri A., Ciapetta P., Delfini R., Romanini L. (1996). Thoracolumbar fractures without neurosurgical involvement: Surgical or conservative treatment?. J. Neurosurg. Sci..

[16] Magerl F., Aebi M., Gertzbein S.D., Harms J., Nazarian S. (1994). A comprehensive classification of thoracic and lumbar injuries. Eur. Spine J..

[17] Denis F. (1983). The three column spine and its significance in the classification of acute thoracolumbar spinal injuries. Spine.

[18] McAfee P.C., Yuan H.A., Fredrickson B.E., Lubicky J.P. (1983). The value of computed tomography in thoracolumbar fractures. An analysis of one hundred consecutive cases and a new classification. J. Bone Jt. Surg.—Ser. A.

[19] McAnany S.J., Overley S.C., Kim J.S., Baird E.O., Qureshi S.A., Anderson P.A. (2015). Open versus Minimally Invasive Fixation Techniques for Thoracolumbar Trauma: A Meta-Analysis. Glob. Spine J..

[20] Divi S.N., Schroeder G.D., Oner F.C., Kandziora F., Schnake K.J., Dvorak M.F., Benneker L.M., Chapman J.R., Vaccaro A.R. (2019). AOSpine—Spine Trauma Classification System: The Value of Modifiers: A Narrative Review with Commentary on Evolving Descriptive Principles. Glob. Spine J..

[21] Rajasekaran S., Kanna R.M., Schroeder G.D., Oner F.C., Vialle L., Chapman J., Dvorak M., Fehlings M., Shetty A.P., Schnake K. (2017). Does the spine surgeon’s experience affect fracture classification, assessment of stability, and treatment plan in thoracolumbar injuries?. Glob. Spine J..

[22] Sebaaly A., Rizkallah M., Riouallon G., Wang Z., Moreau P.E., Bachour F., Maalouf G. (2018). Percutaneous fixation of thoracolumbar vertebral fractures. EFORT Open Rev..

[23] Marcia S., Saba L., Marras M., Suri J.S., Calabria E., Masala S. (2016). Percutaneous stabilization of lumbar spine: A literature review and new options in treating spine pain. BJR Br. J. Radiol..

[24] Marré B., Ballesteros V., Martínez C., Zamorano J.J., Ilabaca F., Munjin M., Yurac R., Urzúa A., Lecaros M., Fleiderman J. (2011). Thoracic spine fractures: Injury profile and outcomes of a surgically treated cohort. Eur. Spine J..

[25] Roy-Camille R., Saillant G., Mazel C. (1986). Plating of thoracic, thoracolumbar, and lumbar injuries with pedicle screw plates. Orthop. Clin. N. Am..

[26] Mobbs R.J., Sivabalan P., Li J. (2011). Technique, challenges and indications for percutaneous pedicle screw fixation. J. Clin. Neurosci..

[27] Phan K., Rao P.J., Mobbs R.J. (2015). Percutaneous versus open pedicle screw fixation for treatment of thoracolumbar fractures: Systematic review and meta-analysis of comparative studies. Clin. Neurol. Neurosurg..

[28] Koreckij T., Park D.K., Fischgrund J. (2014). Minimally invasive spine surgery in the treatment of thoracolumbar and lumbar spine trauma. Neurosurg. Focus.

[29] Lyons R.A., Perry I.M., Littlepage B.N.C. (1994). Evidence for the validity of the short-form 36 questionnaire (SF-36) in an elderly population. Age Ageing.

[30] Singh J., Abrams K.R., Bujkiewicz S. (2021). Incorporating single-arm studies in meta-analysis of randomised controlled trials: A simulation study. BMC Med. Res. Methodol..

[31] Cucherat M., Laporte S., Delaitre O., Behier J.M., d’Andon A., Binlich F., Bureau S., Cornu C., Fouret C., Labouret N.H. (2020). From single-arm studies to externally controlled studies. Methodological considerations and guidelines. Therapies.

