# Supplementary material for: Outcome Measures of Open versus Minimally Invasive Surgery for Thoracolumbar Spinal Traumatic Fractures: A Systematic Review and Meta-Analysis
Source: J Clin Med. 2024 Sep 19;13(18):5558. doi: 10.3390/jcm13185558 (PMC11433229; doi:10.3390/jcm13185558)
Supplement: Supplementary file 1 [file jcm-13-05558-s001.zip › jcm-3059343 - Authorship Change Form.pdf]

Multidisciplinary
Digital Publishing
Institute

***Journal of Clinical Medicine* — Change of Authorship Form**
(Must be Completed and Signed by ALL Authors)

MDPI's Policy

Authors submitting to MDPI journals must ensure that their manuscripts are ethically sound and meet industry-recognized standards that are reflected in MDPI policies (https://www.mdpi.com/ethics). Authors are expected to carefully consider authorship before manuscript submission. Any change to the author list should be made during the editorial process, before manuscript acceptance. Authorship changes, including any addition, removal, or rearrangement of author names will require the approval of all authors including any to be removed. To request any change in authorship, the journal must receive a completed authorship change form that includes the signatures of all authors, and provides a reason for the change.

Any changes to authorship requested after manuscript acceptance will result in a delay in publication. If the manuscript has already been published, requests for a change in authorship will be evaluated and require the publication of a Correction. We reserve the right to request evidence of authorship, and changes to authorship after acceptance will be made at the discretion of MDPI.

1. Manuscript ID: **jcm-3059343**
Manuscript Title: **Outcome Measures of Open versus Minimally Invasive Surgery for Thoracolumbar Spinal Traumatic Fractures: A Systematic Review and Meta-Analysis**

2. Reasons for Authorship Change: Please provide a detailed explanation of the reason for the requested authorship change in the space below. Please note that the Editorial Office will evaluate all post-publication authorship change requests following the Committee on Publication Ethics (COPE) guidelines. We reserve the right to request evidence of authorship, and changes to authorship will be made at the discretion of the Editorial Office. Should a request be approved the update to the authorship list will require the publication of a "Correction". Authors are encouraged to provide any additional materials to support the request in the initial application.

The additional Authors, Andrea Barbanera, Stefania Nape and Sara Lombardi, helped the original Authors in revieweing the responses to the Reviewers

Description of the change: (new author(s) have been added; change in the order of authorship; An author wishes to remove his/her name)

3. Author agreement: The corresponding author should act as a main point of contact and provide details of authorship and author's contributions, although these duties may be delegated to one or more co-authors. Editorial office reserve the right to request signed statement of agreement for the requested change from all listed authors and from the author to be removed or added.

We require consent from all the authors (including from the added/removed co-author) confirming that they are satisfied with the change. Ideally, this will be in the form of an email, preferably from the institution address of the relevant authors

Original Authorship

LIST ALL AUTHORS in the same order as the original (first) submission. For more than 10 use an extra sheet. All authors must sign below agreeing to the Authorship change request and provide handwritten/electronic signature.

Please indicate corresponding author(s) by adding asterisk	Name	Affiliation
author (1)	Felice Esposito	Department of Neurosciences and Reproductive and Dental Sciences, Division of Neurosurgery, Federico II University of Naples, Naples, Italy
author (2)	Ilaria Bove	Department of Neurosciences and Reproductive and Dental Sciences, Division of Neurosurgery, Federico II University of Naples, Naples, Italy
author (3)	Francesca Vitulli	Department of Neurosciences and Reproductive and Dental Sciences, Division of Neurosurgery, Federico II University of Naples, Naples, Italy
author (4)	Andrea Bocchino	Department of Neurosciences and Reproductive and Dental Sciences, Division of Neurosurgery, Federico II University of Naples, Naples, Italy
author (5)	Sara Lombardi	Department of Neurosciences and Reproductive and Dental Sciences, Division of Neurosurgery, Federico II University of Naples, Naples, Italy
author (6)	Giovanni Raffa	Department of Biomedical, Dental and Imaging Sciences, Division of Neurosurgery, University of Messina, Italy
author (7)	Luigi Pintore	Department of Neurosciences and Reproductive and Dental Sciences, Division of Neurosurgery, Federico II University of Naples, Naples, Italy
author (8)	Carmela Palmiero	Department of Neurosciences and Reproductive and Dental Sciences, Division of Neurosurgery, Federico II University of Naples, Naples, Italy
author (9)	Fabrizio Fellico	Department of Neurosciences and Reproductive and Dental Sciences, Division of Neurosurgery, Federico II University of Naples, Naples, Italy
author (10)	Domenico Solari	Department of Neurosciences and Reproductive and Dental Sciences, Division of Neurosurgery, Federico II University of Naples, Naples, Italy
author (11)	Luigi Maria Cavallo	Department of Neurosciences and Reproductive and Dental Sciences, Division of Neurosurgery, Federico II University of Naples, Naples, Italy
author (12)	Teresa Somma	Department of Neurosciences and Reproductive and Dental Sciences, Division of Neurosurgery, Federico II University of Naples, Naples, Italy

New Authorship

All authors must sign below agreeing to the new changes in authorship. The authorship order and appointed corresponding authors must match the new title page of the manuscript. Signatures below certify compliance with the author responsibilities on the next page. List ALL AUTHORS in the same order as the new version.

Please indicate corresponding author(s) by adding asterisk	Name	Affiliation
author (1)*	Felice Esposito	Department of Neurosciences and Reproductive and Dental Sciences, Division of Neurosurgery, Federico II University of Naples, Naples, Italy
author (2)	Ilaria Bove	Department of Neurosciences and Reproductive and Dental Sciences, Division of Neurosurgery, Federico II University of Naples, Naples, Italy
author (3)	Francesca Vitulli	Department of Neurosciences and Reproductive and Dental Sciences, Division of Neurosurgery, Federico II University of Naples, Naples, Italy
author (4)	Andrea Bocchino	Department of Neurosciences and Reproductive and Dental Sciences, Division of Neurosurgery, Federico II University of Naples, Naples, Italy
author (5)	Andrea Barbanera	Division of Neurosurgery, Academic Hospital of Alessandria, Alessandria, Italy
author (6)	Stefania Nape	Department of Neurosciences and Reproductive and Dental Sciences, Division of Neurosurgery, Federico II University of Naples, Naples, Italy
author (7)	Sara Lombardi	Department of Neurosciences and Reproductive and Dental Sciences, Division of Neurosurgery, Federico II University of Naples, Naples, Italy
author (8)	Giovanni Raffa	Department of Biomedical, Dental and Imaging Sciences, Division of Neurosurgery, University of Messina, Italy
author (9)	Luigi Pintore	Department of Neurosciences and Reproductive and Dental Sciences, Division of Neurosurgery, Federico II University of Naples, Naples, Italy
author (10)	Carmela Palmiero	Department of Neurosciences and Reproductive and Dental Sciences, Division of Neurosurgery, Federico II University of Naples, Naples, Italy
author (11)	Fabrizio Fellico	Department of Neurosciences and Reproductive and Dental Sciences, Division of Neurosurgery, Federico II University of Naples, Naples, Italy
author (12)	Domenico Solari	Department of Neurosciences and Reproductive and Dental Sciences, Division of Neurosurgery, Federico II University of Naples, Naples, Italy
author (13)	Luigi Maria Cavallo	Department of Neurosciences and Reproductive and Dental Sciences, Division of Neurosurgery, Federico II University of Naples, Naples, Italy

author (14)	Teresa Somma	Department of Neurosciences and Reproductive and Dental Sciences, Division of Neurosurgery, Federico II University of Naples, Naples, Italy

Authors to be removed (If any)

	name	affiliation	Signature&Date
author (1)			
author (2)			
author (3)			
author (4)			
author (5)			

4. Authors' contributions:

MDPI follows the International Committee of Medical Journal Editors (ICMJE) guidelines on defining the role of authors and contributors. Authorship should be limited to those who have made a significant contribution to the conception, design, investigation, analysis, or interpretation of the reported study. Transparency about the contributions of current authors is encouraged, for example in the form of a CRediT author statement bellow. In addition, authors should have confidence in the integrity of the contributions of their co-authors.

Please list all the author's Contribution here:

Author's Contribution
author (1) Conceptualization, Methodology, Validation, Formal analysis, Resurces, Writing - review & editing, Supervision,
author (2) Validation, Investigation,
author (3) Software, Formal analysis, Writing - original draft,
author (4) Investigation, Writing - original draft,
author (5) Writing - review & editing
author (6) Data curation, Validation
author (7) Validation, Investigation, Data curation, Writing - original draft,
author (8) Validation, Writing - review & editing
author (9) Investigation, Writing - original draft
author (10) Investigation, Data curation, Writing - original draft,
author (11) Investigation, Data curation, Writing - original draft
author (12) Validation, Writing - review & editing
author (13) Resurces, Writing - review & editing
author (14) Conceptualization, Methodology, Resurces, Writing - review & editing, Supervision

Contributor Roles Taxonomy (CRediT)

Conceptualization	Ideas; formulation or evolution of overarching research goals and aims.

Data curation	Management activities to annotate (produce metadata), scrub data and maintain research data (including software code, where it is necessary for interpreting the data itself) for initial use and later re-use.
Formal analysis	Application of statistical, mathematical, computational, or other formal techniques to analyze or synthesize study data.
Funding acquisition	Acquisition of the financial support for the project leading to this publication.
Investigation	Conducting a research and investigation process, specifically performing the experiments, or data/evidence collection.
Methodology	Development or design of methodology; creation of models.
Project administration	Management and coordination responsibility for the research activity planning and execution.
Resources	Provision of study materials, reagents, materials, patients, laboratory samples, animals, instrumentation, computing resources, or other analysis tools.
Software	Programming, software development; designing computer programs; implementation of the computer code and supporting algorithms; testing of existing code components.
Supervision	Oversight and leadership responsibility for the research activity planning and execution, including mentorship external to the core team.
Validation	Verification, whether as a part of the activity or separate, of the overall replication/reproducibility of results/experiments and other research outputs.
Visualization	Preparation, creation and/or presentation of the published work, specifically visualization/data presentation.
Writing - original draft	Preparation, creation and/or presentation of the published work, specifically writing the initial draft (including substantive translation).
Writing - review & editing	Preparation, creation and/or presentation of the published work by those from the original research group, specifically critical review, commentary or revision – including pre- or post-publication stages.

Signature from all the Authors

Name	Signature
Felice Esposito	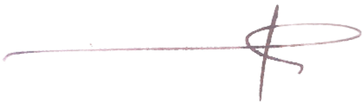
Ilaria Bove	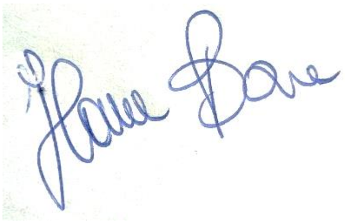
Francesca Vitulli	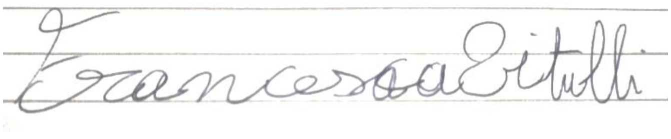

Andrea Bocchino	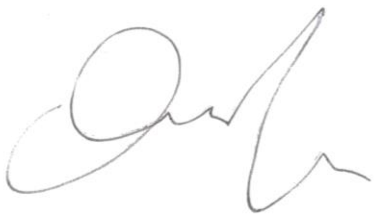
Andrea Barbanera	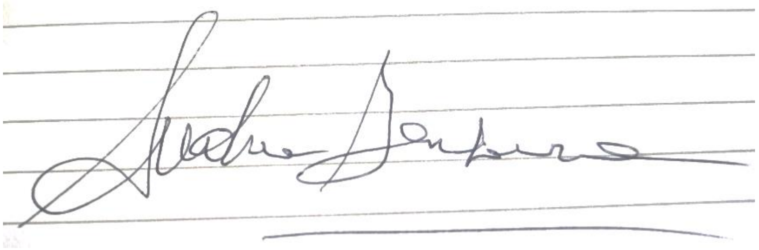
Stefania Nape	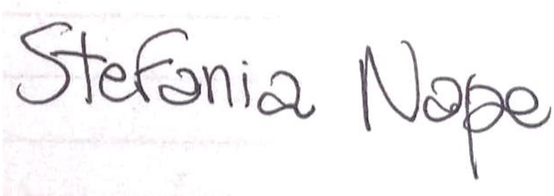
Sara Lombardi	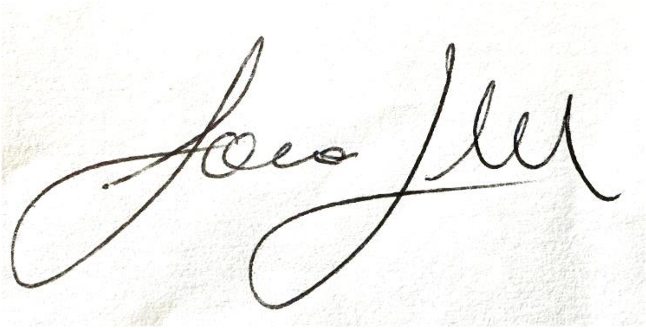
Giovanni Raffa	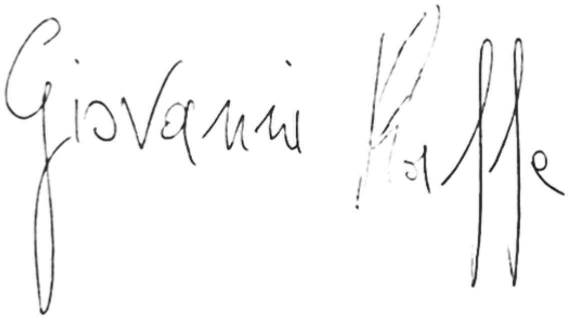

Luigi Pintore	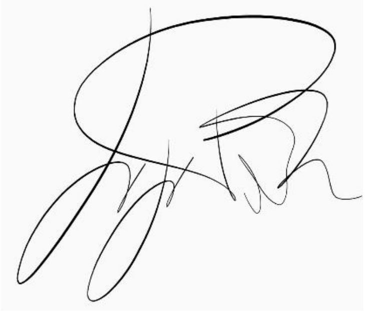
Carmela Palmiero	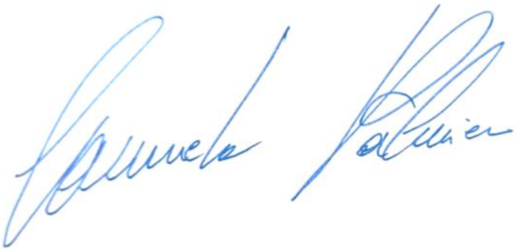
Fabrizio Fellico	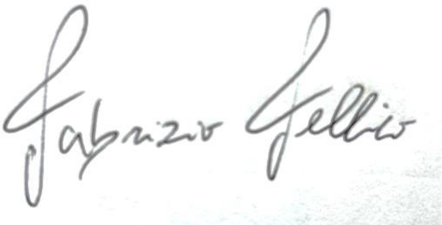
Domenico Solari	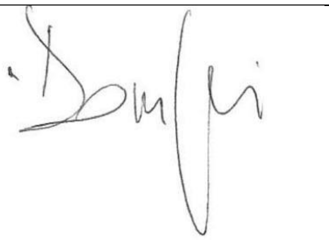
Luigi Maria Cavallo	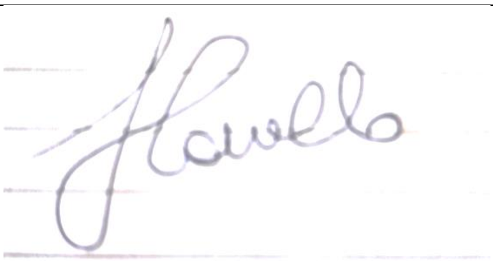
Teresa Somma	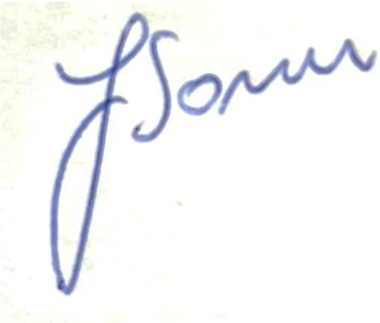
